# Desmopressin as a Treatment in Patients With Von Willebrand Disease: A Systematic Review

**DOI:** 10.7759/cureus.44310

**Published:** 2023-08-29

**Authors:** Andres Beltran, Arturo P Jaramillo, Maria P Vallejo, Luis Acosta, Gabriela Carolina Barberan Parraga, Carlos Luis Guanín Cabrera, Victor G Gaibor, Maria G Cueva

**Affiliations:** 1 General Practice, Universidad Católica de Santiago de Guayaquil, Guayaquil, ECU; 2 General Practice, Universidad Estatal de Guayaquil, Machala, ECU; 3 Internal Medicine, Universidad Católica de Santiago de Guayaquil, Guayaquil, ECU; 4 Internal Medicine, Luis Vernaza Hospital, Guayaquil, ECU; 5 Urology, Universidad Católica de Santiago de Guayaquil, Guayaquil, ECU

**Keywords:** factor viii, bleeding disorders, ddavp, desmopressin, von willebrand diseases

## Abstract

Von Willebrand disease (VWD) and hemophilia A are the most common inherited bleeding disorders. Quantitative or qualitative von Willebrand factor (VWF) anomalies cause this disorder in men and women. VWF, a plasma glycoprotein, relies on platelets for primary hemostasis. It also carries and stabilizes factor VIII in the blood. VWD has several categories. Types 1 and 3 have partial or total VWF quantitative deficiencies. However, type 2 and its subtypes have VWF quality issues. The major treatment is desmopressin (DDAVP), which replaces endogenous VWF and factor VIII (FVIII). Plasma-derived VWF/FVIII products may also be substituted exogenously. Treatment with plasma-derived or recombinant VWF concentrates without FVIII is also possible. The purpose of this retrospective, single-center research was to evaluate DDAVP's efficacy in treating VWD based on many criteria established in the current literature. We looked at the results on Google Scholar, the Cochrane Library, and PubMed/Medline. There were a total of 10 papers found, evaluated, and accepted for inclusion in this study. A comprehensive analysis of DDVAP's role in VWD was compiled from the aforementioned papers. Various aspects of DDVAP were captured by including an analysis of complementary treatments used in surgical and clinical settings. We also describe the treatment's intended impact on the different variations of the disease. Given these results, further investigation is required to determine the most effective method for managing VWD so that it may be included in standard clinical practice.

## Introduction and background

Von Willebrand disease (VWD) is a genetic blood dyscrasia illness characterized by a lack of or deficiency in the von Willebrand factor (VWF). Patients experience mucocutaneous bleeding as well as bleeding following surgery or trauma. As previously reported, VWD has a wide range of phenotypes, including different types from type 1 through type 3 [1,2]. The classification includes VWF antigen, factor VIII (FVIII) activity, VWF activity, and a variety of additional specialized tests. This subclassification has significance for the phenotype of the bleeding and treatment considerations. The treatment of VWD includes using medications that increase FVIII and VWF levels, such as VWF concentrates and desmopressin (DDAVP). Supplemental treatments, such as antifibrinolytics, are also used [3]. Because of its low cost, simple manner of administration, ability to eliminate the need for blood products, and reasonably excellent safety record, DDAVP is an attractive option for providing cover for minor operations and procedures [4]. DDAVP has the capacity to cause the release of VWF and FVIII, resulting in an increase in their levels in the bloodstream. These levels are then used to categorize responses [5]. Because of the variability in responsiveness, it is recommended to evaluate individual patient reactions to DDAVP before using it in a therapeutic setting. Nonetheless, the literature provides inconsistencies in testing indications and definitions of response adequacy. Furthermore, it should be highlighted that there are extant research publications that investigate the use of DDAVP for procedural prophylaxis. However, it is crucial to note that the suggested dose regimens for therapy differ significantly, and there is a dearth of randomized clinical studies undertaken in this field [6-8]. According to the existing evidence, DDAVP seems to be useful for minor procedures and for VWD types 1, 2, and 3, according to various research studies. Other studies described the effect of combining DDVPA and thromboxane during high-bleeding-risk surgeries. We noted that DDVPA showed outstanding hemostasis outcomes in septorhinoplasty in one study. Nonetheless, there is a lack of agreement on the exact description of major and minor procedures [9,10]. In conclusion, there is a paucity of evidence on the impact of DDAVP on periprocedural bleeding outcomes in VWD patients [11]. The different factors all contribute to the increasing difficulty in selecting individuals who may safely be given DDAVP for surgical prophylaxis. The goal of this retrospective study, which was done at a single facility, was to determine the responsiveness of DDAVP using multiple parameters identified in the current literature and to assess the therapeutic effects of using DDAVP in people with VWD [11].

As an example of the use of DDAVP, previous studies have shown that people who have gastrointestinal surgery usually have hypercoagulable disorders, which are characterized by an increased chance of thrombotic events [12]. As a result, it is critical to use considerable care while using hemostatic agents post-surgery [12]. Postoperative anemia has been shown to have a direct association with poor postoperative outcomes, and the usefulness of blood transfusions for patients undergoing gastrointestinal surgery remains a point of contention. Several studies have shown that administering DDAVP may reduce the requirement for blood transfusions while maintaining a similar level of thrombotic event risk [12]. DDAVP, an artificially generated vasopressin derivative, has the capacity to increase factor VIII and VWF secretion, improve platelet function, and reduce blood loss during surgical operations. Nonetheless, DDAVP's ambiguous effects on urine volume and renal function are a direct outcome of its antidiuretic qualities. There have been few clinical studies on the effect of DDAVP on hemostasis after gastrointestinal surgery [12]. The most important point of this study is going into the potential of DDAVP acetate in reducing blood loss in people who had significant hemorrhage during gastrointestinal surgery, as well as its effects on antidiuretic functions after gastrointestinal surgery [12].

## Review

Methodology

We did a systematic evaluation using free full-length papers and the Preferred Reporting Items for Systematic Reviews and Meta-Analyses (PRISMA) to describe our approach and results from three different databases: PubMed, the Cochrane Library, and Google Scholar were consulted for the screening of the papers, which was done through techniques like MeSH keyword and Boolean search.

Study Duration

This review started on July 1, 2023.

Search Strategy

A detailed examination of it can be found in the Appendix.

Eligibility Criteria and Study Selection

To assess eligibility, two investigators carefully read the full title and content of each paper. We selected the latest literature and articles published in the past five years, including papers written in the English language, or if the full free-text English-language translation is available. Articles were excluded if the full text of the papers could not be retrieved. Articles focusing on DDAVP as a treatment in VWD were strictly chosen. Gray literature and proposal papers were also not included.

Data Management

Two independent writers evaluated papers based on titles and abstracts. Following that, significant abstracts were examined for a complete, free full-text examination. A third author evaluated the research after evaluating the chosen studies, if there was any disagreement. Information from the relevant publications was then collected. The first author's name, type, year of publication, study design, and results were taken as priorities. Finally, duplicates were deleted.

Quality Assessment

We used the Assessment of Multiple Systematic Reviews (AMSTAR) form and the Cochrane risk of bias assessment tools for clinical trials and for systematic reviews and meta-analyses.

Results

Search Results

**Figure 1:**
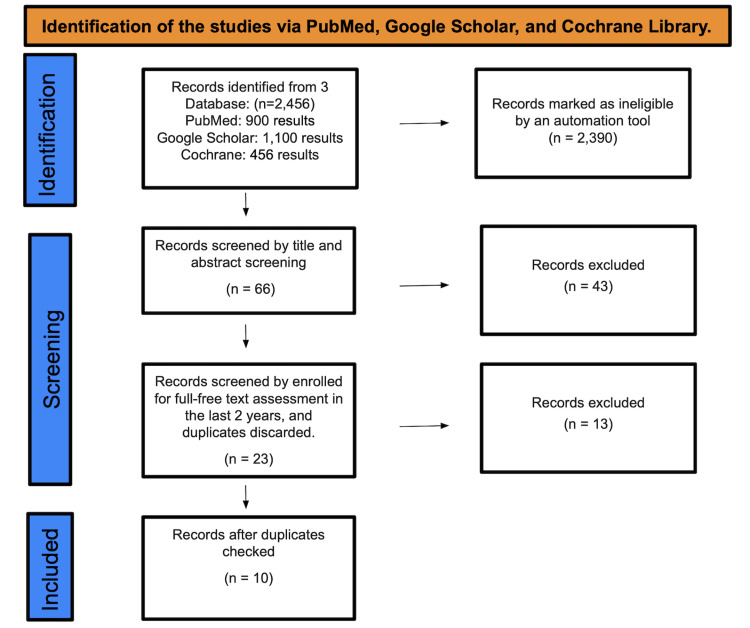
Identification of studies via databases and registers

A total of 2,456 studies were found after searching PubMed, Google Scholar, and the Cochrane Library. A total of 2,390 were marked as ineligible by an automation tool. There were a total of 66 studies that underwent title and abstract screening, with 43 papers being discarded. The remaining 23 papers were chosen by full-free text evaluation in the previous five years, and after discarding duplicates, resulting in the elimination of 13 studies, only 10 studies were enlisted for the final collection of data. Figure 1 depicts the detailed PRISMA flow diagram of the article selection procedure.

An in-depth description of the articles is given in Table 1.

**Table 1:** Table of data extraction AMSTAR: Assessment of Multiple Systematic Reviews; RCT: randomized clinical trial; SRL and MA: systematic review literature and meta-analysis; VWD: von Willebrand disease; DDAVP: desmopressin; PMP: platelet microparticle; PK: pharmacokinetic; PAC-1: protease-activated receptor 1; IBD: inherited bleeding disease.

Author	Year of publication	Study design	Quality tool	Primary research	Outcome evaluation
Du et al. [13]	2023	SRL	AMSTAR checklist	The expected outcomes were VWD prevalence, incidence, mortality, burden of disease, patient characteristics, and current therapeutic management and treatments.	Mucocutaneous symptoms such as epistaxis, menorrhagia, and oral or gum bleeding were reported in 72%-94% of VWD patients.
Chandrakumaran et al. [11]	2023	SRL	AMSTAR checklist	Examined documents pertaining to patients with VWD aged 18 years who experienced DDAVP challenge testing from 2007 to 2022.	The effectiveness of DDAVP prophylaxis periprocedural seems to be evident in VWD patients.
Akbarpour et al. [14]	2023	RCT	Cochrane risk of bias assessment tool	A double-blinded randomized controlled trial (RCT) was carried out between the years 2021 and 2022, encompassing individuals ranging from 18 to 40 years of age.	There was no statistically significant difference (p > 0.05) in operational time between the three groups. No significant results were found when the effect of the study group was evaluated on systolic blood pressure, diastolic blood pressure, and pulse rate.
Persyn et al. [15]	2022	RCT	Cochrane risk of bias assessment tool	Included in the study were 15 out of 18 patients who underwent tests using DDAVP. They had factor VIII deficiency, or VWD, which caused them to suffer greatly.	Only minimal changes were seen in P-selectin and PAC-1 binding. PMP patients fall into two categories. Following DDAVP medication, PMP levels in the first cohort of six people increased by 120%. The second cluster, seven persons, did not change PMPs after DDAVP.
Kalot et al. [16]	2022	SRL	AMSTAR checklist	We looked through MEDLINE, EMBASE, and CENTRAL from their inceptions through August of this year.	The review provided estimates of sensitivity and specificity, which were utilized to simulate diagnostic strategies and offer evidence-based recommendations for a clinical practice guideline.
Heijdra et al. [17]	2022	RCT	Cochrane risk of bias assessment tool	This study aims to assess its prognostic powers. PK models were used in several scenarios: 1) DDAVP testing with a sample size of at least 30; 2) medical procedures with 70 participants, 30 of whom received DDAVP, 30 VWF-containing concentrates, and 10 a combination of both; and 3) bleeding episodes with 20 participants, 10 of whom received desmopressin.	For this study, a two-sided p-value of less than 0.05 is considered significant. Number and timing of DDAVP infusions will be quantified.
Akbarpour et al. [18]	2022	RCT	Cochrane risk of bias assessment tool	The research included participants over the age of 18. Participants were randomly given a low-dose or high-dose intranasal DDAVP or a fake therapy 1 hour before general anesthesia.	The results showed no differences between low-dose DDAVP and a placebo. Blood loss differed significantly between the high-dose DDAVP and control groups.
Franchini et al. [19]	2021	SRL	AMSTAR checklist	PubMed and Medline electronic databases were scoured using a haphazard method to find complete-text publications regarding the immediate and prolonged preventive treatment of VWD.	The objective of prophylaxis in the long term is to hinder hemorrhaging in individuals who have an increased likelihood of recurrent and unanticipated hemorrhaging in the joints, nasal passages, and digestive system.
de Azevedo Kinalski et al. [20]	2021	SRL	AMSTAR checklist	The selected studies focused on individuals with IBDs (such as hemophilia A or B or VWD) and examined minor and major oral procedures in the fields of medicine and dentistry.	A total of 4,152 citations were meticulously examined, out of which 257 were deemed suitable for inclusion in the ultimate analysis.
Wang et al. [12]	2020	SRL	AMSTAR checklist	An RCT was conducted for landmark research. The research included carefully chosen gastrointestinal surgery patients from three top institutions. These patients were randomly separated into two groups for impartial outcomes.	Between June 1, 2015, and June 1, 2017, 59 patients participated. A day after the procedure, the DDAVP group had a lower hemoglobin decline than the normal saline group.

Discussion

The purpose of this SRL is to provide the findings of DDAVP in patients diagnosed with VWD, with a specific focus on the various clinical and surgical contexts represented by the publications that were compiled. It is vital to note that some articles were not included in the research process; hence, this has been a limitation of our SRL. This should be emphasized as an important point. As a result of the aforementioned, we discovered that Du et al. examined various treatment options for VWD with various types and degrees of symptoms and, interestingly, discovered that only a small proportion of VWD patients are receiving long-term VWF prophylaxis [13]. Long-term prevention was mostly given to people with type 3 VWD to prevent bleeding in the joints or GI system. According to the current VWD treatment standards, people who have had serious and regular bleeding in the past and have VWD should be given longer-term prevention. On the other hand, the team that made the recommendations said that there is not enough strong proof to show that continued prevention is good for the health of this particular group [13]. DDAVP, replacement therapy with VWF antifibrinolytics, and blood transfers were some of the methods that could be used. There were 17 sources that talked about the use of temporary or conditional prevention, like when surgery is being done or when a baby is being born. In addition, there are nine studies that go into detail about long-term prevention. Plasma-derived VWF, DDAVP, VWF/FVIII concentrates, recombinant VWF, and thromboxane (TXA) were some of the choices for temporary or conditional prevention [13]. In different studies, people who were given long-term prevention for VWD had different ages, sexes, and types of VWD. The usual age was between 15.9 and 60 years old, and most of the people were men. VWD type 1 was found in 3.1% to 12.5% of people, type 2 was found in 17.6% to 46.9% of people, and type 3 was found in 40.6% to 74.5% of people. In a total of eight studies, the reasons for long-term prevention were discussed. From these studies, it was found that joint bleeding happened in about 13% to 43.8% of cases, while epistaxis, or bleeding from the mouth, happened in about 23% to 34.4% of people. Also, gastric bleeding was found in a wide range of people with VWD, from 6.3% to 63.6%. Lastly, about 5% to 21.9% of women with VWD had heavy monthly vaginal bleeding [13]. The study by Chandrakumaran et al. [11] showed that 84 out of 94 cases responded well to DDAVP. Their set conditions, which required a VWF activity/factor VIII amount of at least 0.50 IU/mL after 1 hour, led them to that answer. Still, the number of people who reacted positively to DDAVP ranged from 53.2% to 91.5%, which is pretty amazing. During the time of the study, a total of 99 surgeries were done, and DDAVP was used before each one. Eighty-six treatments, or 86.7% of the cases, were done using only DDAVP, with or without the addition of TXA [11]. Only 31 of these treatments were rated as "major," while the other 55 were rated as "minor." For people with VWD, using DDAVP alone or in combination with TXA as a way to prevent bleeding during surgery has been shown to be very effective. Also, it has been seen that putting in place pre-procedural preventive treatments that include DDAVP could lower the number of bleeding problems after major medical procedures. Additionally, people with type 2 VWD can use this approach [11]. In a study by Akbarpour et al. [14], it was found that bleeding during an open septorhinoplasty surgery can cause problems and affect the quality of the operating field in a bad way. This means that clinical results from traditional methods might not be good enough. Because of this, there is an immediate need to use drug treatments with a longer half-life and better effectiveness. It has been suggested that putting DDAVP on the skin could help with open septorhinoplasty because it could reduce blood loss at the surgery site [11]. In the setting of septorhinoplasty, this randomized controlled trial (RCT) looked at how different doses of external DDAVP affected postoperative bleeding and the surgical field. The effects of this finding could be very important in the field of rhinoplasty, where less blood loss could mean less swelling and bleeding of the soft tissues. So, this makes it easier for the working surgeon to make a more accurate assessment [11].

In the study by Persyn et al. [15], it was found that when DDAVP was given, the levels of VWF and FVIII went up. This was noticed in a group of nine people with VWD and eight people with hemophilia A. These factors increased by an average of 3.3% and 3.1%, respectively, in people with VWD and hemophilia A [15]. The aim of the study was to determine whether platelet microparticles (PMPs) started to form after DDAVP was injected into living organisms. In the study, it was found that the 13 patients who were tested released an average of 37% more PMPs than they did before. This rise was noticed exactly 1 hour after the DDAVP was given [15]. DDAVP seems to speed up the growth of coat platelets, which are triggered when collagen and thrombin work together. Platelets are covered with proteins that help blood clot and molecules that hold cells together [15]. Also, the results given here back up what Horstman et al. found in their study about PMPs, where they saw a rise in the number of PMPs released in about 27% of the people they looked at in a period of 60 minutes [21]. Trummer et al. also saw an increase in PMPs after giving DDAVP to people with type 1 VWD or an unclear bleeding tendency, and the results presented here are very similar to what they found [15,22]. In a different study, Kalot et al. [16] looked at the accuracy of tests used to find and describe people with VWD. The evidence went from low to middling in terms of how sure it was. This has shown that the VWF:Ag ratio, VWF propeptide, and DDAVP trial can be used to show that the VWF propeptide/VWF:Ag ratio and the VWF:Ag half-life have a negative relationship [16]. According to the findings, identifying people with type 1 VWD who have a high plasma VWF level can be done simply by looking at the steady-state fraction of plasma VWF propeptide and VWF [16]. In a different study done by Akbarpour et al. [18], a significant link was found between the type of surgery and the amount of blood loss. However, it is important to note that a mean difference of only 0.4 ml was found between the low-dose desmopressin group and the control group [18]. But when compared to the high-dose DDAVP group, the controls saw a 29.6 ml drop in bleeding during surgery. The size of the resulting effect is a very important factor [18]. At both the first and second time points, the link between the type of surgery and the quality of the operating field was seen. Due to the low quality of the data in this area, we still do not know how DDAVP affects overall blood loss. Also, previous studies have shown that people who have septorhinoplasty may lose less blood if they are given desmopressin [18].

Based on research done by Franchini et al., intravenous infusion of DDAVP at a dose of 0.3 µg is recommended as a diagnostic tool for figuring out how best to use the drug in a clinical setting [19]. A good response is one in which the average activity of VWF goes up by at least two times and both VWF and factor VIII keep going up. When it comes to cases of VWD other than type 1, DDAVP cannot be used in every situation. In type 2A, the drug does not work as well because the VWF levels are high, but the function is still low. Recent studies have found that about 2 million people have a wide range of reactions. It is important to remember that in many situations, giving DDAVP is seen as inappropriate and not advised. In type 2N, the medicine usually makes up for the lack of FVIII, but it only stays active in the system for a short time [19]. VWD needs long-term prevention mostly when it causes serious bleeding, like hemarthrosis with target joints and arthropathy. When it comes to people like this, who usually have VWD type 3 and, to a lesser extent, serious VWD type 1, it may be better to use a plan of long-term protection instead of only treating bleeding when it happens [19]. de Azevedo Kinalski et al. did a groundbreaking study that gives us a new way of looking at inherited blood diseases (IBDs) [20]. Over the past 100 years, there have been huge changes in how these disorders are treated. Notably, the development of factor preparations that include clotting factors has led to huge improvements in how people with IBD are treated and their total life expectancy [20]. Even though most people with IBD only use factor concentrates occasionally, there are some cases where people need to take clotting factors on a regular basis as a preventative measure because their condition is so bad, mostly to stop bleeding in their muscles and joints [20]. Even though most people with IBD only get factor preparations when they need them, there are some people who need clotting factors given to them all the time as a preventative measure. This is mostly because of how bad their situation is, and the main goal is to stop as much muscle and joint loss as possible [20].

## Conclusions

We got different outcomes from the different studies. Our first conclusion was about the different types of VWD. In some studies, VWF was used only in people with VWD type 3 and blood problems in the GI tract and joints. For this, long-term DDAVP prophylaxis was used. Another conclusion related to IBD was that multiple pharmacological treatments were used, among them VWF, DDAVP, VWF/FVIII concentrates, etc. The second conclusion taken is that in some procedures, DDAVP has good outcomes as a monotherapy, but in other moderately bleeding-risk procedures, it has been DDAVP + TXA that has had excellent outcomes. Our third conclusion is that in the surgical field, due to the high risk of bleeding, especially in one of the articles that was chosen, taking septorhinoplasty as an example, high-quality outcomes are achieved with the use of DDAVP for hemostasis. Our fourth conclusion related to the high stimulation of coagulation promoters after receiving DDAVP in patients with VWD in most cases indicates its use in type 2N VWD. After all that was mentioned, we agreed that DDAVP has a fundamental role in the treatment of VWD, but that it is good to take into account which kind of procedure is needed and how the combination of other treatments can enhance its outcome. Thus, more studies on which specific VWD type is the best option for the use of DDAVP with or without the combination with other treatments should be given.

## Appendices

Search strategy

PubMed, Google Scholar, and Cochrane library were used to collect database using the following: (("von Willebrand Diseases/blood"[Majr] OR "von Willebrand Diseases/complications"[Majr] OR "von Willebrand Diseases/diagnosis"[Majr] OR "von Willebrand Diseases/diet therapy"[Majr] OR "von Willebrand Diseases/drug therapy"[Majr] OR "von Willebrand Diseases/physiopathology"[Majr] OR "von Willebrand Diseases/prevention and control"[Majr] OR "von Willebrand Diseases/rehabilitation"[Majr] OR "von Willebrand Diseases/therapy"[Majr] OR "von Willebrand Diseases/urine"[Majr])) AND ("von Willebrand Diseases/blood"[Majr:NoExp] OR "von Willebrand Diseases/complications"[Majr:NoExp] OR "von Willebrand Diseases/diagnosis"[Majr:NoExp] OR "von Willebrand Diseases/diet therapy"[Majr:NoExp] OR "von Willebrand Diseases/drug therapy"[Majr:NoExp] OR "von Willebrand Diseases/physiopathology"[Majr:NoExp] OR "von Willebrand Diseases/prevention and control"[Majr:NoExp] OR "von Willebrand Diseases/rehabilitation"[Majr:NoExp] OR "von Willebrand Diseases/therapy"[Majr:NoExp] OR "von Willebrand Diseases/urine"[Majr:NoExp]) AND (("von Willebrand Factor/adverse effects"[Majr] OR "von Willebrand Factor/agonists"[Majr] OR "von Willebrand Factor/analogs and derivatives"[Majr] OR "von Willebrand Factor/antagonists and inhibitors"[Majr] OR "von Willebrand Factor/chemistry"[Majr] OR "von Willebrand Factor/pharmacokinetics"[Majr] OR "von Willebrand Factor/pharmacology"[Majr] OR "von Willebrand Factor/therapeutic use"[Majr])) AND ("von Willebrand Factor/adverse effects"[Majr:NoExp] OR "von Willebrand Factor/agonists"[Majr:NoExp] OR "von Willebrand Factor/analogs and derivatives"[Majr:NoExp] OR "von Willebrand Factor/antagonists and inhibitors"[Majr:NoExp] OR "von Willebrand Factor/chemistry"[Majr:NoExp] OR "von Willebrand Factor/pharmacokinetics"[Majr:NoExp] OR "von Willebrand Factor/pharmacology"[Majr:NoExp] OR "von Willebrand Factor/therapeutic use"[Majr:NoExp])
